# Association of *LAG3* genetic variation with an increased risk of PD in Chinese female population

**DOI:** 10.1186/s12974-019-1654-6

**Published:** 2019-12-17

**Authors:** Wenyuan Guo, Miaomiao Zhou, Jiewen Qiu, Yuwan Lin, Xiang Chen, Shuxuan Huang, Mingshu Mo, Hanqun Liu, Guoyou Peng, Xiaoqin Zhu, Pingyi Xu

**Affiliations:** 1 Department of Neurology, The First Affiliated Hospital of Guangzhou Medical University, Guangzhou, 510120 China; 2 Department of Physiology, School of Basic Medical Sciences, Guangzhou Medical University, Guangzhou, 511436 China

**Keywords:** LAG3, Parkinson disease, α-Synuclein, Transmission, Biomarker

## Abstract

**Background:**

Emerging evidence suggests that α-synuclein (α-syn) aggregation and intercellular transmission contributes to pathogenesis of Parkinson’s disease (PD) and the toxic fibrillary α-syn binds lymphocyte-activation gene 3 (LAG3) receptor that mediates α-syn transmission. The deletion of LAG3 in animal models was shown to limit α-syn spreading and alleviate the pathological changes of dopaminergic neurons and animal behavioral deficits induced by α-syn aggregation. However, little is known about the genetic association of LAG3 variation with human PD development.

**Objective:**

Here we investigated LAG3 single nucleotide polymorphisms (SNPs) and examined the levels of soluble LAG3 (sLAG3) of CSF and serum from Chinese PD patients.

**Methods:**

We enrolled 646 PD patients and 536 healthy controls to conduct a case-control study. All the participants were genotyped using Sequenom iPLEX Assay and the partial cerebrospinal fluid (CSF) and serum samples were assessed by Meso Scale Discovery electrochemiluminescence (MSD-ECL) immunoassay to measure sLAG3 concentration.

**Results:**

As a result, distributions of rs1922452-AA (1.975, 95% confidence interval (CI) 1.311–2.888, *p* = 0.001) and rs951818-CC (OR = 2.03, 95% CI 1.369–3.010, *p* = 0.001) genotype frequencies were found higher in the female PD patients than controls, respectively, and a strong linkage disequilibrium (LD) was calculated on the variants. The level of sLAG3 in CSF of PD patients was found to significantly differ from that of controls (51.56 ± 15.05 pg/ml vs 88.49 ± 62.96 pg/ml, *p* < 0.0001). Meanwhile, the concentration of α-synuclein in CSF of patients was significantly lower than that of controls (939.9 ± 2900 pg/ml vs 2476 ± 4403 pg/ml, *p* < 0.0001) and the level of sLAG3 was detected to be positive correlation with that of α-synuclein in the control group (*r* = 0.597, *p* = 0.0042), but not in PD group (*r* = 0.111, *p* = 0.408).

**Conclusion:**

In summary, our data suggested that LAG3 SNPs increase the PD risk of Chinese female population and the sLAG3 may be a potential biomarker predicted for PD development.

## Introduction

Parkinson’s disease (PD) is the *second* most common neurodegenerative disease that affects 1% of individuals aged > 60 in the world [1]. Intracellular inclusion bodies in neurons which comprise primarily of misfolded or aggregated α-synuclein are the neuropathological hallmark of the disease [2]. But the etiology of PD is still not understood although genetic and environmental factors play vital roles in PD development [3].

Lymphocyte-activation gene 3 (LAG3) gene maps to chromosome 12p13 and mainly regulates the immune system as major histocompatibility complex (MHC) class II ligand evolutionarily related to cluster of differentiation 4 (CD4) T lymphocytic reaction [4, 5]. A recent study showed that α-synuclein transmission by the pattern of neuron-to-neuron could be initiated by LAG3 dysfunction. As a transmembrane protein, LAG3 binds α-syn preformed fibrils (PFFs) with high affinity and initiates α-syn PFF endocytosis, transmission, and toxicity in cells. Moreover, LAG3^−/−^ mice delayed the α-syn PFF-induced loss of dopamine neurons and presented animal biochemical and behavioral changes [6]. Based on high-resolution magnetic resonance images scanned for the brain of PD patients, significant select cortex atrophy was detected in the higher LAG3 expressional regions compared to the gene lower expressional areas [7]. Recent studies also indicated that immune dysfunction is required for the progression of PD initiated by α-synuclein aggregation [8]. This extracellular α-syn can activate microglia to initiating an inflammatory response. CD4+ lymphocytes invading the brain play an important role in the progress of PD. [9] A series of complements and cytokines, such as IL-1, IL-2, IL-6, and TNF-α and IFN-γ, have been changed in the peripheral blood and cerebrospinal fluid (CSF) of PD patients [10]. A disorder of peripheral lymphocyte subsets in PD patients was also reported [11]. More than others, α-syn fragments could be specifically identified and presented to T cells to induce toxic T cell response in the peripheral blood cells of PD patients, which has a similar feature with autoimmune disease [12]. As an immune regulator LAG3 may be involved in the neuroinflammatory mechanisms of PD pathogenesis by medicating the transmission of α-syn with a “prion”-like pattern.

Till now, there was no investigation to explore the possible influence of LAG3 variants on the Chinese PD population, although there were three variants of LAG3 such as rs1922452, rs951818, and rs870849 were identified as a potential risk factor to multiple sclerosis (MS) [13, 14]. Here we aimed to investigate the possible association between LAG3 variants and PD in a Chinese PD population. It is worth noting that soluble LAG3 (sLAG3) is generated from the alternative splicing of LAG3 or shed from the cell surface [15, 16]. The serum sLAG level was reported to be associated with the PD development [17], but no data was indicated to the association of sLAG3 with PD pathogenesis with CSF examination. Thus, we measured the level of sLAG3 and α-syn in partial CSF of PD patients and controls.

## Material and methods

### Participants and genomic DNA analysis

A total of 1182 subjects were enrolled in this study, including 646 PD patients and 536 controls. PD patients fulfilled the clinical diagnosis of PD according to the UK Parkinson Disease Society Brain Bank criteria. The exclusion criteria for the control group were tumors, infections, autoimmune diseases, and other neurodegenerative diseases. All samples were obtained from the Departments of Neurology of the first affiliated hospital at Guangzhou Medical University from 2014 to 2017. DNAs were purified under the standard protocols provided by Blood DNA Kit manufacturer (Tiangen Biotech, Beijing, China) and the genotyping was analyzed at the Beijing Genomics Institute (BGI) Co., Ltd. (Beijing, China).

### Ethics approval

All the subjects wrote their agree-statement forms and the project was approved by the Ethics Committee of the first affiliated hospital of Guangzhou Medical University.

### sLAG3 MSD-ECL

CSF samples were collected from 21 healthy controls and 58 patients with PD. Serum samples were obtained from 61 healthy controls and 78 PD patients. Those with a history of tumors, infections, autoimmune diseases, and other neurodegenerative diseases were excluded, as these diseases may affect sLAG3 levels. While blood tubes were centrifuged at 3000 rounds per minute for 10 min at 4 °C, the serum was separated into collection tubes. CSF and serum samples were aliquoted and stored at − 80 °C. Analyzers were blinded to all serum and CSF samples and participants’ information. The levels of sLAG3 and α-synuclein were measured using the Meso Scale Discovery electrochemiluminescence (MSD-ECL) immunoassay. All the samples were run in duplicate.

### Data analysis

Data statistics were carried out by SPSS, version 21 and GraphPad Prism version 6. Student’s *t* tests or Mann-Whitney *U* test was used to assess the difference of continuous variables between PD and control groups. Other variables for gender, genotype and allele distribution were analyzed with chi-squared (*χ*^2^) test or Fisher’s exact test. The diagnostic performance was assessed by the AUC of the ROC curve. Cutoff values were calculated using sensitivity and specificity that maximized Youden’s index. The correlations were evaluated using linear regression analysis (Spearman’s correlation). *p* < 0.05 was considered significant. The statistical power was calculated using QUANTO software version 1.2.4 (Additional file 5).

## Results

### Allele distribution of LAG3 in the Chinese population

The PD group contained 387 men and 259 women with an average of 63.40 ± 12.67 years, and the male-female ratio is consistent with the epidemiological incidence of PD. The control group composed of 341 males and 195 females with an average of 63.73 ± 10.07 years. There was no significant difference in demographics between PD patients and controls (Table 1).

**Table 1:** The demographic characterization of PD patients and controls

Samples	Demographics	Control	PD	*p*
DNA	Number	536	646	
	Gender (male/female)	341/195	387/259	0.191^b^
	Age years (mean ± SD)	63.73 ± 10.08	63.40 ± 12.67	0.622^a^
CSF	Number	21	58	
	Sex (male/female)	12/9	31/27	0.80^b^
	Age, years(mean ± SD)	53.90 ± 19.27	56.86 ± 9.43	0.36^a^
	H&Y score		2.01(1–3)	
	Disease duration		3.25(0.5–11)	

*PD* Parkinson’s disease, *SD* standard deviation, *H&Y* Hoehn & Yahr

^a^*p* Value obtained by t test, ^b^*p* Value obtained by χ2 test.

^a^*p* value obtained by t test

^b^*p* value obtained by *χ*^2^ test

**Fig. 1:**
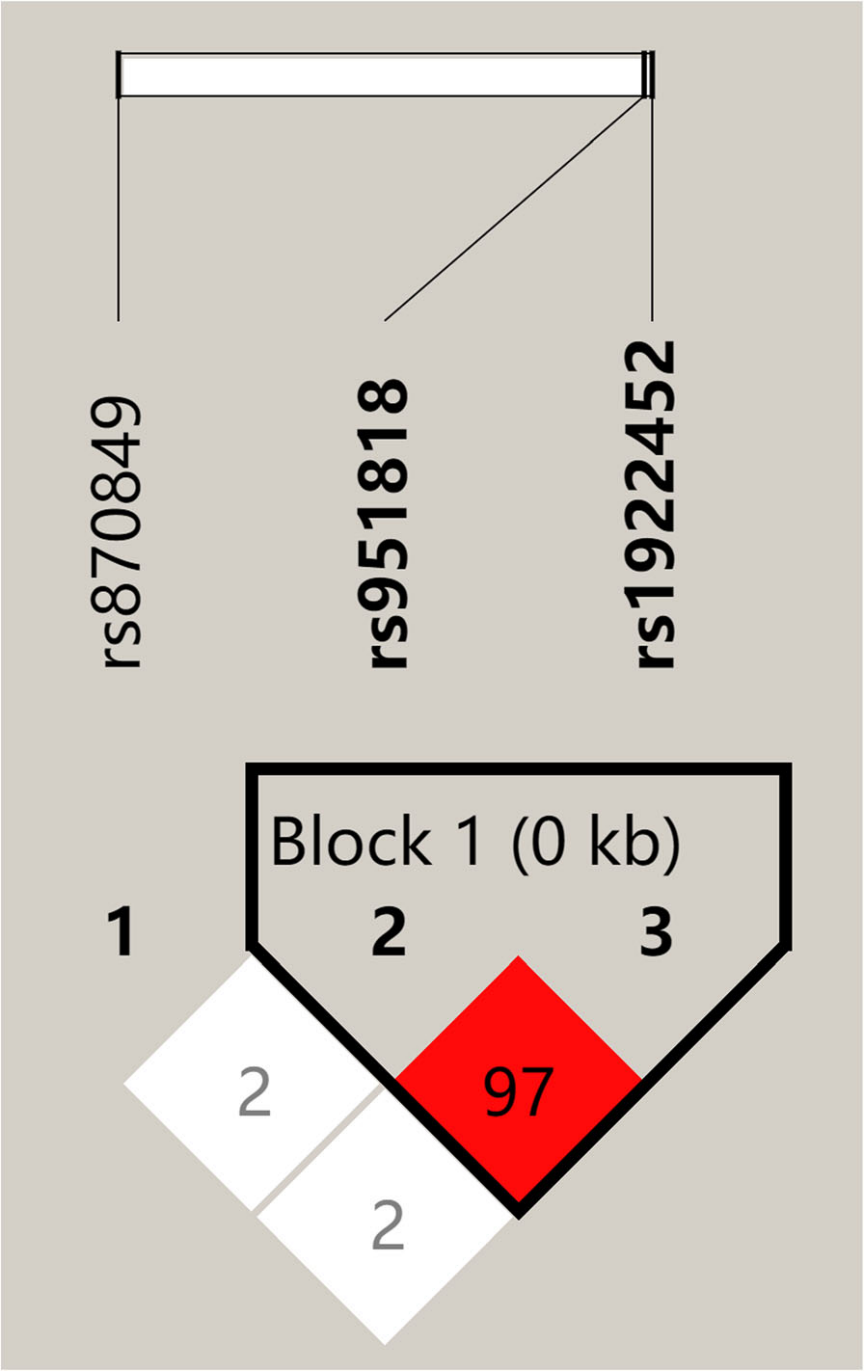
Linkage disequilibrium (|*D*′|) between pairs of SNPs in LAG3. Between two of the SNPs, the measure of *D*′ was shown graphically according to color, where deep color represents high *D*′. rs1922452 and rs951818 were found to be in LD with each other, |*D*′| = 0.97,which the *D*′ value was shown in square

We have genotyped three SNPs of LAG3 gene and tested them for the association of PD in a case-control study consisting of 646 PD patients and 536 controls. The genotype and allele distribution of rs870849, rs1922452, and rs951818 were very similar in both of patient and control groups. Statistical testing with *χ*^2^ showed non-significant *p* values by the allelic associational analysis with a dominant, negative model (Table 2). In consideration of gender as an important factor of PD development, we carried out an in-depth stratification analysis with gender on the LAG3 SNPs on each group. As a result, the genotype of rs1922452-AA and rs951818-CC was found to be significantly higher in female PD patients than in controls, respectively (rs1922452, OR = 1.975, *p* = 0.001; rs951818 OR = 2.03, *p* = 0.001, Table 2). It is worthy to note that the two SNPs are only 137 base pairs apart and present a strong LD score with one other (*D*′ = 0.974, Fig. 1).

**Table 2:** The analysis of genotype and allele distribution on PD cases and controls

SNP	Models	PD	Control	OR	*p*
rs951818	Dominant ((CC + CA)/AA)	552/77	460/72	1.122 (0.795–1.583)	0.570
	Recessive (CC/(CA + AA))	282/347	213/319	1.217 (0.963–1.538)	0.113
	Allele (C/A)	834/424	673/391	1.143 (0.963–1.356)	0.137
Female	Dominant ((CC + CA)/AA)	218/33	154/40	1.716 (1.036–2.843)	0.048
	Recessive (CC/(CA + AA))	118/133	59/135	2.030 (1.369–3.010)	0.001
	Allele (C/A)	336/166	213/175	1.663 (1.266–2.185)	0.000
Male	Dominant ((CC + CA)/AA)	334/44	306/32	0.794 (0.491–1.284)	0.412
	Recessive (CC/(CA + AA))	164/214	154/184	0.916 (0.682–1.230)	0.610
	Allele (C/A)	498/258	460/216	0.906 (0.727–1.130)	0.414
rs1922452	Dominant ((AA+AG)/GG)	555/79	466/59	0.889 (0.621–1.274)	0.583
	Recessive (AA/(AG + GG))	282/352	208/317	0.945 (0.703–1.271)	0.108
	Allele (A/G)	837/431	674/376	1.083 (0.913–1.286)	0.384
Female	Dominant ((AA+AG)/GG)	222/34	165/25	0.989 (0.568–1.722)	1
	Recessive (AA/(AG + GG))	118/138	58/132	1.975 (1.311–2.888)	0.001
	Allele (A/G)	340/172	223/157	1.392 (1.058–1.831)	0.022
Male	Dominant ((AA+AG)/GG)	333/45	301/34	0.836 (0.521–1.340)	0.531
	Recessive (AA/(AG + GG))	164/214	150/185	1.221 (0.965–1.544)	0.766
	Allele (A/G)	497/259	451/219	0.932 (0.747–1.162)	0.568
rs870849	Dominant ((CC + CT)/TT)	615/16	513/11	0.824 (0.379–1.792)	0.698
	Recessive (CC/(CT + TT))	441/189	373/151	0.945 (0.732–1.218)	0.708
	Allele (C/T)	1056/205	886/162	0.942 (0.752–1.179)	0.641
Female	Dominant ((CC + CT)/TT)	246/4	184/5	1.671 (0.443–6.310)	0.508
	Recessive (CC/(CT + TT))	186/64	136/53	1.133 (0.740–1.734)	0.643
	Allele (C/T)	432/68	320/58	1.151 (0.788–1.682)	0.527
Male	Dominant ((CC + CT)/TT)	369/12	329/6	0.561 (0.208–1.511)	0.339
	Recessive (CC/(CT + TT))	255/125	237/98	0.844 (0.614–1.160)	0.333
	Allele (C/T)	624/137	566/104	0.837 (0.633–1.106)	0.229

*PD* Parkinson’s disease, *OR* odds ratio, *95% CI* 95% confidence interval. *p* value obtained by continuity corrected *χ*^2^ test. The genotype of rs19922452-AA and rs951818-CC was found to be significantly higher in female PD patients than in controls, respectively (rs951818, OR = 2.03, *p* = 0.001; rs19922452, OR = 1.975, *p* = 0.001)

### Analysis on the relationship of sLAG3 with the risk of PD

**Fig. 2:**
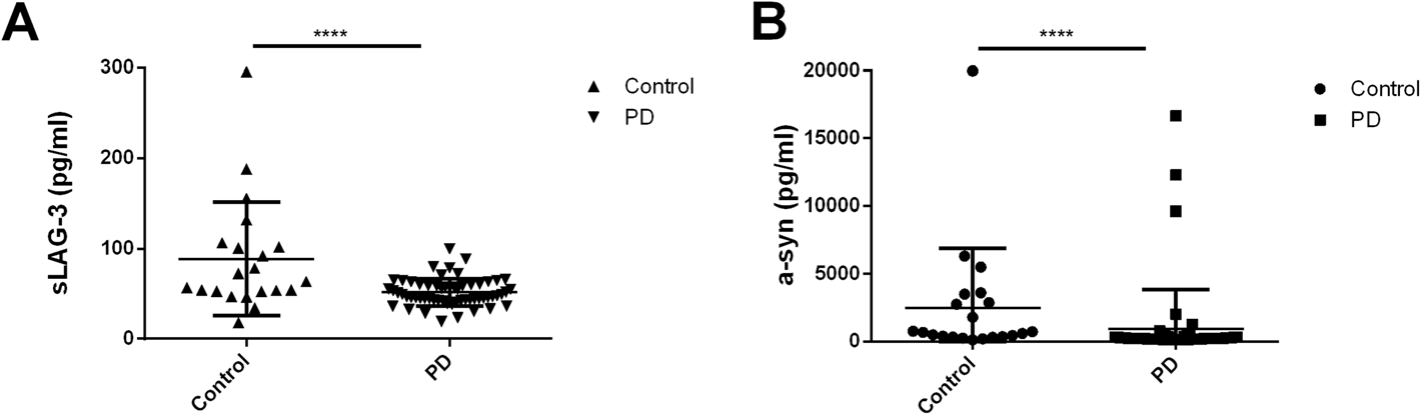
sLAG3 and α-synuclein levels in CSF. **a** sLAG3 levels of CSF in control subjects (88.49 ± 62.96 pg/ml, *N* = 21) and PD patients (51.56 ± 15.05 pg/ml, *N* = 58) by MSD-ECL examination. Data represented as mean ± SD, *p* < 0.0001. **b** α-synuclein concentration in CSF of controls (2476 ± 4403 pg/ml, N = 21) and PD patients (939.9 ± 2900 pg/ml, *N* = 58) by MSD-ECL measurement. Data represented as mean ± SD, *p* < 0.0001.

**Fig. 3:**
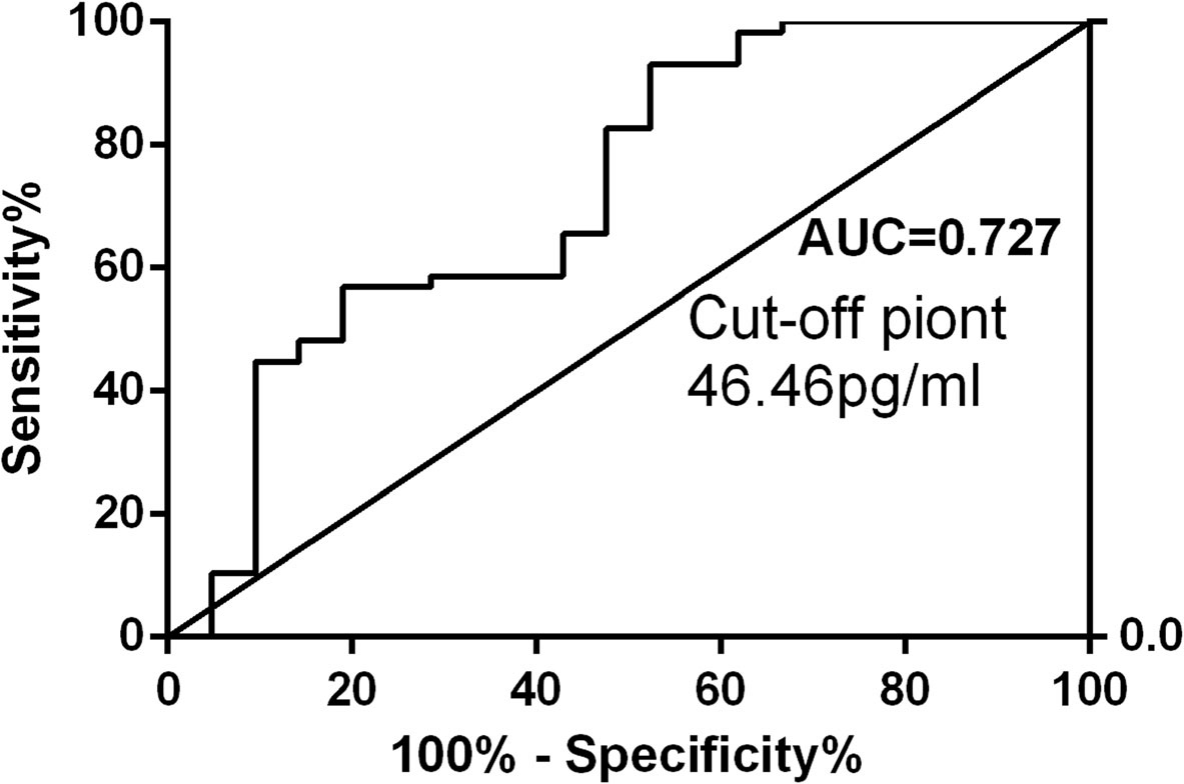
The analysis of receiver operating characteristic (ROC) curves on sLAG3 of CSF from PD patients. ROC curves were made by the Youden’s index using the maximum of sensitivity + specificity – 1. AUC = area under the curve. sLAG3 could be a potential biomarker for PD prediction

To explore the relationship between sLAG3 and the risk of PD, we measured sLAG3 contents of CSF collected from partial PD patients and controls (Table 1). In consideration of only 71.4% sLAG3 in CSF specimens were detected by ELISA with a sensitivity of 6.25 pg/ml, we decided the examination of sLAG3 and α-synuclein of CSF preferred to MSD-ECL measurement (Additional file 1). As a result, the mean value of sLAG3 in CSF was significantly lower in PD patients compared to HC subjects (PD, 51.56 ± 15.05 pg/ml; control 88.49 ± 62.96 pg/ml, *p* < 0.0001), but no difference of serum sLAG3 was found in the two groups (PD, 433.2 ± 25.67, *N* = 78; control 485.0 ± 29.67 pg/ml, *N* = 61; *p* = 0.19, Additional file 2). Although LAG3 variations were found to be associated with female PDs, no significant difference in CSF sLAG3 were detected between male and female patients (Additional file 3). Meanwhile, the levels of sLAG3 in CSF was not in correlation with serum sLAG3 in PD patients(*r* = − 0.317, *p* = 0.186, *N* = 20, Additional file 4). The α-syn concentration of CSF was significantly decreased in PD patients compared to that in controls (PD 939.9 ± 2900 pg/ml, *N* = 58; control 2476 ± 4403 pg/ml; *N* = 21, *p* < 0.0001, Fig. 2). To evaluate whether sLAG3 could be the potential biomarker for PD risk, we performed a ROC curve analysis by Youden’s index maximums for its sensitivity and specificity. As showed in Fig. 3, the AUC value of sLAG3 was 44.64 pg/ml with a cutoff threshold of 0.727 following a sensitivity and higher specificity calculated as 44.83% and 90.48%, respectively.

**Fig. 4:**
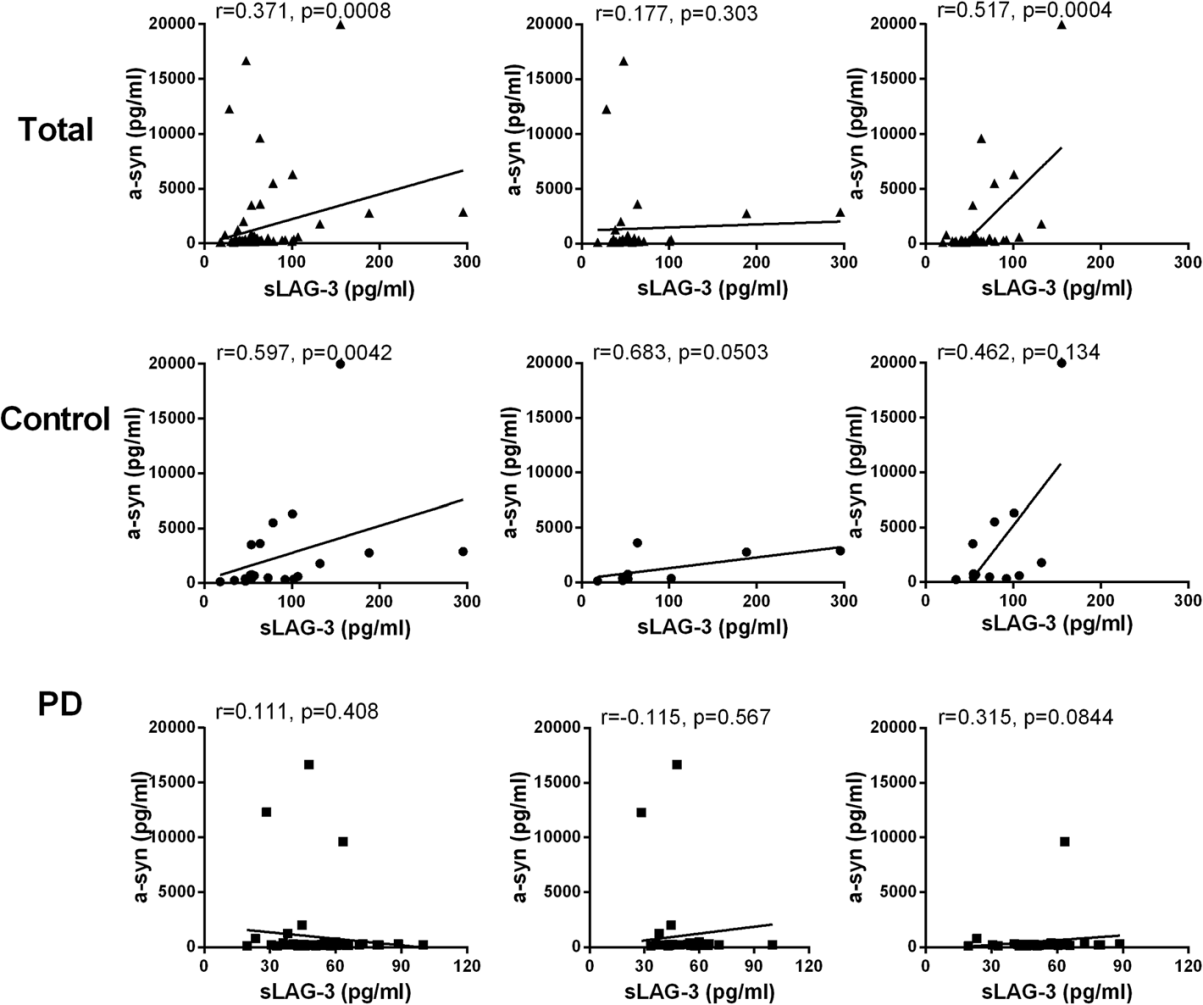
The correlation analysis of α-synuclein and sLAG3 in CSFs from patients and controls. The levels of sLAG3 in CSF was positively correlated with the concentration of α-synuclein in controls (*r* = 0.597, *p* = 0.0042)

In consideration of LAG3 mediating α-syn intercellular transmission among neurons, we decided to investigate the correlation between sLAG3 and α-synuclein in CSFs. The levels of sLAG3 was potentially positively correlated with α-synuclein in the control group (*r* = 0.597, *p* = 0.0042, Fig. 4), but not in the PD group (*r* = 0.111.*p* = 0.408). Since LAG3 variations were confirmed to be associated with female PDs, we next analyzed the correlation between the level change of sLAG3 and that of α-syn in CSF on males and females separately. As a result, no correlation was detected on male and female patients or controls (Additional file 3). Moreover, the concentration of sLAG3 in CSF has no correlation with age, PD’s H-Y stage, or disease duration (*r* = 0.07, *p* = 0.694; *r* = 0.140, *p* = 0.429; and *r* = 0.088, *p* = 0.442, respectively, data not show; Additional file 4).

## Discussion

In this study, three LAG3 SNPs were genotyped at first time in a case-control cohort of Chinese patients with PD. Interestingly, rs1922452-AA and rs951818-CC genotypes were verified to increase the risk of female PD and the levels of sLAG3 were significantly decreased in CSF as a potential biomarker of PD development, especially in female patients.

Generally, gender and aging are the important factors to affect the development of PD because of hormones and genetic susceptibility contributing to the neural immune function [18–20]. Physically, females with age over 50 years accompany a decreased of estrogen in menopause [21]. It was reported that microglia become reactive and upregulate the expression of MHC I and II in the forebrain of postmenopausal women who more easily suffered from immune disorders [22, 23]*.* As an immune regulator, LAG3 have a high affinity with MHC-II molecules to play a role in multiple autoimmune disorders [24]. By suppressing microglia and astrocyte activation via MHC-I presentation, estrogen modulates neural inflammation procession in the central nervous system [25, 26]. As more, the inflammatory response was confirmed in dopaminergic nigrostriatal system which is increasingly recognized in PD pathogenesis [27]. Thus, it is reasonable to speculate that LAG3 variants will increase the risk of PD in female population under an immune dysfunction condition.

LAG3 can splice into two different mRNAs to encode transmembrane protein LAG3 and alternative splicing soluble protein sLAG3 [13]. Interestingly, the two types of protein play different roles to modulate the immune system. The membrane protein LAG3 negatively regulates T cell function [28] involving in suppressing central nervous system autoimmunity. Blockade and cleavage of membrane LAG3 can augment T cell proliferation and cytokine production in vitro and inhibit the suppressive activity of Treg cells in vitro and in vivo [29, 30]. Although alternative spliceosome sLAG3 functions as an immune adjuvant enhance anti-tumor T cell function response to an irradiated tumor cell vaccine [29], surface-shed sLAG3 blocked the recruitment of antigen-presenting cells by reducing the differentiation of monocytes into scavenging macrophages or antigen-presenting dendritic cells [31]. In conclusion, the balance of LAG3 and sLAG3 in the central nervous system (CNS) jointly maintains the immune homeostasis. It is reasonable to speculate that high expression of LAG3 Treg cells from PD patients induces neural immune dysfunction which becomes to be easily attacked by aggregated α-synuclein. It worth to note that rs1922452 and rs951818 localize at RNA-seq intron-spanning reads; the two variants could disrupt the RNA splice frame to affect the expression of LAG3 and the product of sLAG3, disturb neural immune balance, and result in α-synuclein transmission among neurons. Furthermore, sLAG3 (containing D1–D4) can also be shed from the cell surface via proteolytic cleavage of ADAM10 and ADAM17 [32]. Many reports revealed that the enzyme activity of ADAM17 and ADAM10 decreased in AD which shares similar pathological mechanism with PD pathogenesis [33–35] and could cause a decline of sLAG3 in PD patients’ CSF.

There was a report about an increase of serum sLAG3 in PD patients [17]. However, paradoxically, no difference of normalized serum sLAG3 between PD patients and controls was found out in our study (Additional file 2). One possible explanation might mainly account for the technological platform because MSD-ECL assays may have higher sensitivity, repeatability, homogeneity, and stability than ELISA assays in micro-protein measurement.

Although it is the first time to find sLAG3 as a potential marker in CSF [36], our data only revealed that the level of sLAG3 is potentially positively correlated with a concentration of α-synuclein in the control CSF samples, and not in the PD population. LAG3 can bind with α-synuclein to facilitate protein transmission [6], but the cell-to-cell transmission of α-syn is thought to associate with other mechanisms. Firstly, α-syn may be released in exosomes/secretory vesicles, consequently taken up by endocytosis through heparan sulfate proteoglycans (HSPGs) [37, 38]. Secondly, α-syn oligomers can directly penetrate the plasma membrane through pore-like structures [39]. Thirdly, tunneling nanotubes (TNTs) where F-actin interacts with the plasma membrane to form membrane bridges between the cells can mediate the spread of α-syn [40]. Meanwhile, sLAG3 is either the partial product of LAG3 cleavage generated by alternative splicing or can be shed from the cell surface LAG3 [15, 16], which only partially stands for the interaction between LAG3 and α-syn. Above all, it is reasonable to explain why the change of sLAG3 does not reflect on the situation of α-synuclein in PD patients’ CSF.

## Conclusion

Our data revealed an association of rs1922452 and rs951818 at *LAG3* with an increased risk of PD in the Chinese female population. sLAG3 was significantly lower in the CSF of PD patients than that in healthy controls, and might be suggested as a potential biomarker for the prediction of PD development.

## Supplementary information

**Additional file 1: Figure S1.** CSF sLAG-3 levels measured by ELISA and MSD-ECL in PD patients. Red bar: ECL; Blue bar: ELISA.

**Additional file 2: Figure S2.** Alterations in serum sLAG-3 levels in patients with PD and controls. sLAG-3 levels of serum in control subjects (585.7 ± 595.4 pg/ml *N* = 61) and PD patients (532.5 ± 284.3 pg/ml, *n* = 78) by MSD-ECL examination. Data represented as mean ± SD, *p* = 0.484.

**Additional file 3: Figure S3.** Alterations in CSF sLAG-3 levels in male and female patients and controls. A: The concentration of CSF sLAG-3 in female subjects (62.41 ± 48.81 pg/ml, *N* = 36) and male subjects (60.51 ± 26.61 pg/ml, *n* = 43 by MSD-ECL examination. Data represented as mean ± SD, *p* = 0.358. B: sLAG-3 levels of CSF in female control subjects (96.29 ± 89.48 pg/ml, *N* = 9) and PD patients (82.64 ± 36.00 pg/ml, *N* = 12) by MSD-ECL examination. Data represented as mean ± SD, *p* = 0.411. C: sLAG-3 levels of CSF in female control subjects (51.12 ± 14.63 pg/ml, *N* = 27) and PD patients (51.94 ± 15.63 pg/ml, *N* = 31) by MSD-ECL examination. Data represented as mean ± SD, *p* = 0.617.

**Additional file 4: Figure S4.** Correlation of CSF sLAG-3 with serum sLAG-3.

**Additional file 5. **The statistical power of the sample. The power was greater than 0.99, *p* = 0.000015.

## Data Availability

All manuscripts reporting the results of these clinical trials excluding individual de-identified participant data will be shared; related data will be shared; related documents such as the study protocol and statistical analysis plan will be shared; and data will be available to all interested researchers upon request.

## Abbreviations

α-syn: α-Synuclein
CD4: Cluster of differentiation 4
CNS: Central nervous system
LAG3: Lymphocyte-activation gene *3*
LD: Linkage disequilibrium
MHC: Major histocompatibility complex
MS: Multiple sclerosis
MSD-ECL: Meso Scale Discovery electrochemiluminescence
PD: Parkinson’s disease
PFFs: Preformed fibrils
sLAG3: Soluble LAG3
SNPs: Single nucleotide polymorphisms
