# Supplementary figures and images for: Association of LAG3 genetic variation with an increased risk of PD in Chinese female population
Source: J Neuroinflammation. 2019 Dec 17;16:270. doi: 10.1186/s12974-019-1654-6 (PMC6918662; doi:10.1186/s12974-019-1654-6)

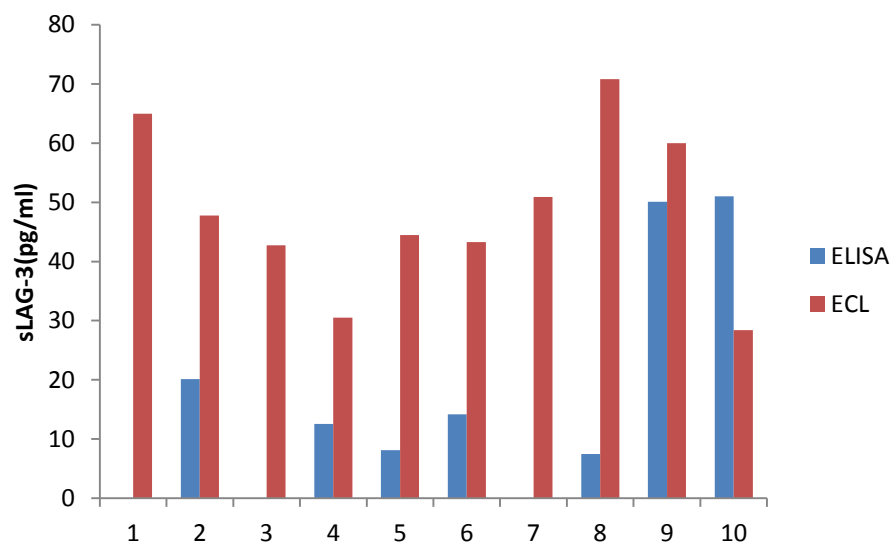

Supplement: Supplementary file 1 — Additional file 1: Figure S1. CSF sLAG-3 levels measured by ELISA and MSD-ECL in PD patients. Red bar: ECL; Blue bar: ELISA. [file 12974_2019_1654_MOESM1_ESM.pdf]

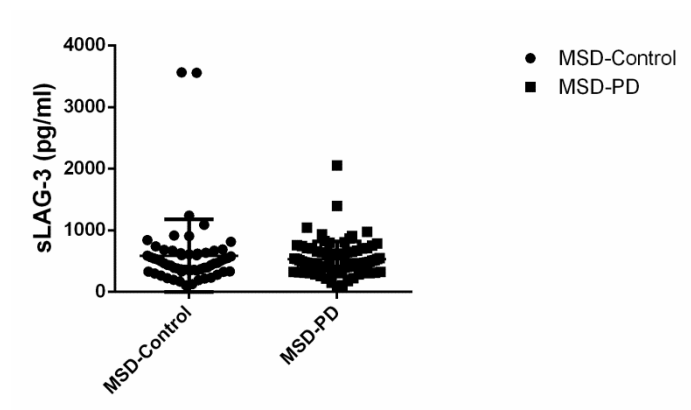

Supplement: Supplementary file 2 — Additional file 2: Figure S2. Alterations in serum sLAG-3 levels in patients with PD and controls. sLAG-3 levels of serum in control subjects (585.7 ± 595.4 pg/ml N = 61) and PD patients (532.5 ± 284.3 pg/ml, n = 78) by MSD-ECL examination. Data represented as mean ± SD, p = 0.484. [file 12974_2019_1654_MOESM2_ESM.pdf]

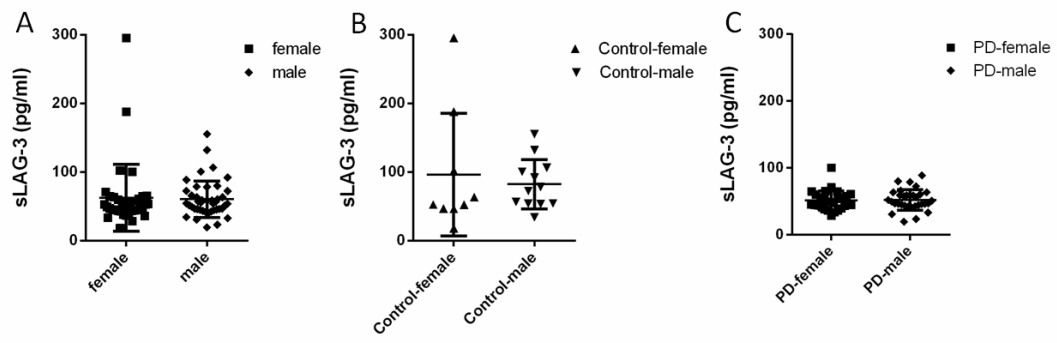

Supplement: Supplementary file 3 — Additional file 3: Figure S3. Alterations in CSF sLAG-3 levels in male and female patients and controls. A: The concentration of CSF sLAG-3 in female subjects (62.41 ± 48.81 pg/ml, N = 36) and male subjects (60.51 ± 26.61 pg/ml, n = 43 by MSD-ECL examination. Data represented as mean ± SD, p = 0.358. B: sLAG-3 levels of CSF in female control subjects (96.29 ± 89.48 pg/ml, N = 9) and PD patients (82.64 ± 36.00 pg/ml, N = 12) by MSD-ECL examination. Data represented as mean ± SD, p = 0.411. C: sLAG-3 levels of CSF in female control subjects (51.12 ± 14.63 pg/ml, N = 27) and PD patients (51.94 ± 15.63 pg/ml, N = 31) by MSD-ECL examination. Data represented as mean ± SD, p = 0.617. [file 12974_2019_1654_MOESM3_ESM.pdf]

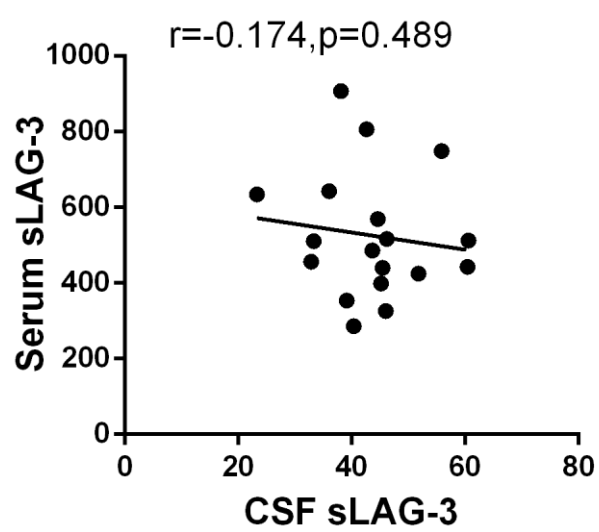

Supplement: Supplementary file 4 — Additional file 4: Figure S4. Correlation of CSF sLAG-3 with serum sLAG-3. [file 12974_2019_1654_MOESM4_ESM.pdf]
