# Supplementary material for: Association of LAG3 genetic variation with an increased risk of PD in Chinese female population
Source: J Neuroinflammation. 2019 Dec 17;16:270. doi: 10.1186/s12974-019-1654-6 (PMC6918662; doi:10.1186/s12974-019-1654-6)
Supplement: Supplementary file 5 — Additional file 5. The statistical power of the sample. The power was greater than 0.99, p = 0.000015. [file 12974_2019_1654_MOESM5_ESM.pdf]

Outcome:	Disease
Design:	Matched case-control
Hypothesis:	Gene only
Sample size:	1182 case-control pairs
Significance:	0.050000, 2-sided
Gene	
Mode of inheritance:	Recessive
Allele frequency:	0.8070
Disease model	Summary parameters
$*P_0$ 0.000015	k_P 0.000020
R_G: 1.5000	(*indicates calculated value)

Parameter	Null	Full	Reduced
Gene	$\beta_G=0$	β_G	——

	Power	
R_G	Gene	P_0
1.5000	0.9947	0.000015
1.7500	0.9999	0.000013
2.0000	0.9999	0.000012
2.2500	0.9999	0.000011
2.5000	0.9999	0.000010
2.7500	0.9999	0.000009
3.0000	0.9999	0.000009
